# Using amyloid PET imaging to diagnose Alzheimer’s disease in patients with multiple sclerosis

**DOI:** 10.1007/s00415-020-09969-z

**Published:** 2020-06-18

**Authors:** Magdalena Kolanko, Zarni Win, Neva Patel, Omar Malik, Christopher Carswell, Anastassia Gontsarova, Richard Nicholas, Richard Perry, Paresh Malhotra

**Affiliations:** 1 Department of Brain Sciences, Faculty of Medicine, Imperial College London, London, W6 8RP UK; 2 Department of Neurology, Imperial College Healthcare NHS Trust, London, UK; 3 Department of Nuclear Medicine, Imperial College Healthcare NHS Trust, London, UK; 4 Department of Radiology, Imperial College Healthcare NHS Trust, London, UK; 5 UK Dementia Research Institute, Imperial College London, London, UK

**Keywords:** Alzheimer’s disease, Multiple sclerosis, Dementia, PET imaging

## Abstract

**Background:**

Cognitive dysfunction affects 40–60% of individuals with multiple sclerosis (MS). The neuropsychological profile commonly consists of a subcortical pattern of deficits, although a proportion of patients have a severe progressive cortical dementia. However, patients with MS can be affected by other neurodegenerative diseases, such as Alzheimer’s disease (AD). Little is known about the co-existence of these two conditions but distinguishing dementia due to MS alone from a coexisting neurodegenerative disease is challenging. Amyloid PET imaging has allowed improved AD diagnosis, especially in patients with atypical presentations or multiple possible causes of cognitive impairment. Amyloid PET demonstrates increased cortical signal in AD, whereas reductions in subcortical uptake are associated with demyelination. To the authors knowledge, there are no reports of clinical Amyloid PET use in MS patients with dementia.

**Methods:**

Here, three MS patients presenting to the Cognitive Neurology Clinic with progressive cognitive impairment are described. Due to lack of diagnostic clarity from standard investigations, they underwent Amyloid PET Imaging with ^18^F-florbetapir according to established appropriate use criteria and after review by a multidisciplinary team.

**Results:**

Two patients were diagnosed with AD based on positive Amyloid PET imaging and were subsequently started on cholinesterase inhibitor treatment. The other patient had a negative scan, leading to further investigations and identification of another potential cause of worsening cognitive impairment.

**Conclusions:**

The experience from this case series suggests that Amyloid PET Imaging may be of diagnostic value in selected patients with MS and dementia. In these individuals, it may provide diagnostic clarity and assist with therapeutic decisions.

## Introduction

As the population ages and the treatment of Multiple Sclerosis (MS) advances, more individuals with MS will develop age-related neurodegenerative disorders, including Alzheimer’s disease (AD) [10]. However, diagnosing AD in cognitively impaired MS patients is challenging and little is known about the coexistence of the two conditions [9].

Cognitive dysfunction is common in MS, affecting 45–65% of patients [14]. Neuropsychological symptoms include deficits in processing speed, executive function, episodic memory and visuospatial function. However, progressive dementia with prominent amnesia and classic cortical features has also been described [19]. Grey matter involvement and cortical tissue loss in MS are increasingly recognised [4] and correlate better with MS-related cognitive impairment than white matter lesion load [2]. Deep grey matter and cortical atrophy appears to be tightly coupled to cognitive decline in late relapsing–remitting and progressive MS [7], and hence, distinguishing this from coexisting Alzheimer’s disease using structural imaging alone can be problematic.

The introduction of two reliable biomarkers for amyloid pathology, cerebrospinal fluid amyloid β1-42/tau levels and amyloid PET Imaging (API), has transformed pre-mortem diagnosis in AD, particularly in patients with atypical presentations or co-morbidities known to impair cognition. Clinical API utilises fluorinated tracers (^18^F-florbetapir, ^18^F-florbetaben or ^18^F-flutemetamol) that bind to amyloid beta in cerebral amyloid plaques, leading to increased cortical signal in AD. There is a growing research interest in using amyloid PET as a surrogate marker of MS demyelination–remyelination, and reductions in white matter tracer uptake have been demonstrated with demyelination in MS [3]. However, this is independent of cortical β-amyloid deposition, and tracer uptake in the cortex in late MS has been found to be no different from age-matched controls [21].

Two recent studies have examined the association between amyloid PET tracer binding and cognitive function in MS [11, 21], and a prospective population-based study used amyloid PET to investigate beta-amyloid accumulation in ageing MS patients and matched controls [20]. Yet, there are no reports of API to diagnose AD in patients with established MS and increasing cognitive impairment.

Here, three MS patients presenting with progressive cognitive impairment are described. Each had API because of a lack of diagnostic clarity with standard dementia investigations. It is highlighted how clinical diagnosis of AD might be aided by this approach.

## Methods

### Patients

Patients presented to the Cognitive Neurology clinic at Charing Cross Hospital, London, between 2016 and 2019. Each was referred with progressive cognitive impairment and had an established diagnosis of MS.

### Decision to investigate with amyloid PET imaging

All patients were first clinically assessed by an experienced cognitive neurologist before structural imaging was discussed at a Cognitive Neurology and Radiology Multidisciplinary meeting. The decision to perform API was made by consensus among neuroradiologists, nuclear medicine specialists and cognitive neurologists, according to appropriate use criteria proposed by the Amyloid Imaging Taskforce (AIT) [8].

### (18)F-florbetapir imaging

Images were qualitatively read as amyloid positive or negative by two nuclear medicine radiologists, in accordance with that outlined in the Amyvid summary of product characteristics (Eli Lilly, 2012), which had been approved by the FDA in 2012 and EMA in 2013. Positive scans had two or more brain areas of reduced or absent grey–white differentiation, or one or more areas in which grey matter activity was intense and clearly exceeded activity in adjacent white matter.

## Results

### Case 1

**Fig. 1:**
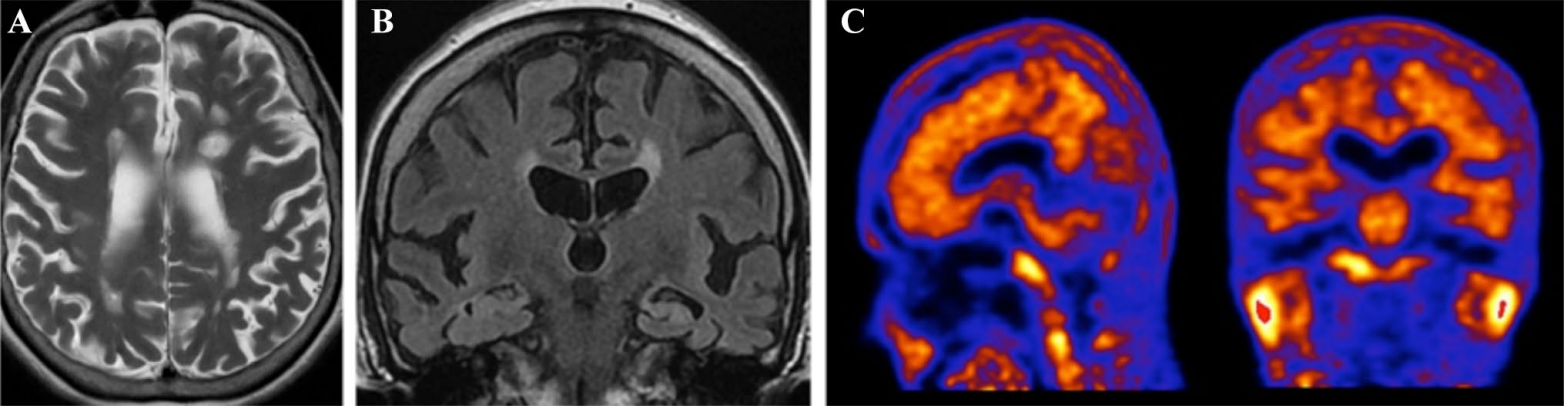
Case 1. Axial T2-weighted (**a**) and coronal FLAIR (**b**) images through the brain showing typical MS lesions in the periventricular white matter, perpendicular to the ependymal surface. Note generalised neuroparenchymal loss, with relative sparing of the temporal lobe white matter (left mesial temporal atrophy score is 2; right mesial temporal atrophy score is 1). Sagittal and coronal amyloid PET images (**c**) demonstrating generalised increased activity within the cortical grey matter in all lobes with complete loss of grey–white differentiation consistent with widespread amyloid deposition

A woman presented with a 6-year history of progressive amnesia and language impairment that had started in her mid-60s, followed by a rapid deterioration over a year with urinary incontinence and self-neglect. She was initially diagnosed by a Memory Clinic with dementia secondary to MS. However, a second opinion was sought in view of her fairly preserved physical function but rapid worsening in cognition. She had been diagnosed with MS in 1994 and MRI imaging at the time had been consistent with demyelination. She had not had any major relapses since 1997 and was not on disease modifying treatment. There was a family history of dementia with the patient’s sister being diagnosed in her late 60s. On examination, she had severe cognitive impairment, with difficulty completing the Mini Mental State Examination. Brain MRI showed a number of inflammatory demyelinating lesions (Fig. 1a), as well as moderate global cerebral volume loss (Fig. 1b). API, carried out following MDT discussion, was reported as positive (Fig. 1c). The patient was given a diagnosis of AD and started on a cholinesterase inhibitor, but continued to decline cognitively.

### Case 2

**Fig. 2:**
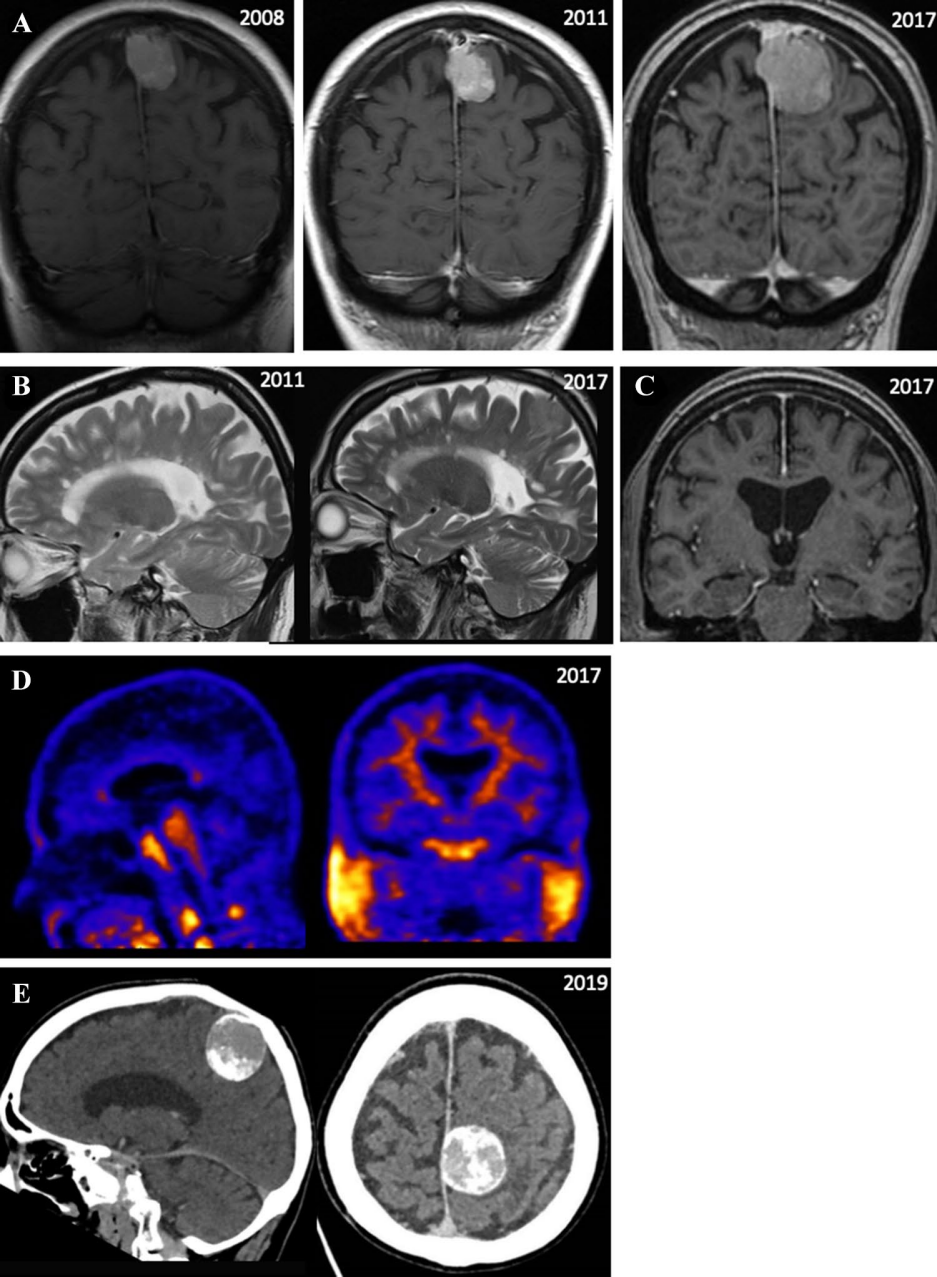
Case 2. **a** Post-contrast coronal T1-weighted images through the brain showing growth of the meningioma in the left parietal region. Meningioma volumes: 5.5 cc in 2008, 5.5 cc in 2011, 21 cc in 2017. **b** Sagittal T2-weighted MRI images demonstrating stable lesion load in the periventricular white matter between 2011 and 2017. **c** Coronal post-contrast T1-weighted MRI image demonstrating normal bilateral hippocampal volumes at age 76. **d** Negative amyloid PET scan, with clear differentiation between grey and white matters and absence of tracer uptake in the cerebral grey matter. **e** CT scan showing an interval increase in size of the left parasagittal meningioma (4.3 × 3.7 × 3.5 cm) with associated local mass effect and vasogenic oedema

A woman in her mid-70s presented with a 5-year history of gradual cognitive decline with progressive amnesia, inattention, organisational difficulties and spatial disorientation. She was diagnosed with relapsing–remitting MS in 1994 and had also been under observation for a parafalcine meningioma since 2008 (Fig. 2a). ACE (Addenbrookes Cognitive Examination)-III score was 87/100, and neuropsychological assessment showed inefficiencies in inhibition and switching, non-verbal abstract reasoning, processing speed and free recall of structured auditory information. Performance was intact on tasks of language, visuospatial ability, recall of unstructured auditory information, visual memory, simple attention and working memory. Brain MRI showed MS changes with little progression when compared to previous scans (Fig. 2b). The meningioma was noted to have evolved over time with slight increase in size but without associated oedema (Fig. 2a). API was negative (Fig. 2d), and it was felt that she had transitioned to secondary progressive MS with associated cognitive decline. In view of worsening cognition (ACE-III score 75/100 (from 87/100)), repeat imaging was performed, which showed an interval increase in meningioma size with worsening oedema (Fig. 2e), potentially contributing to her cognitive impairment. She was referred to the neurosurgical team who decided to keep her under regular review.

### Case 3

**Fig. 3:**
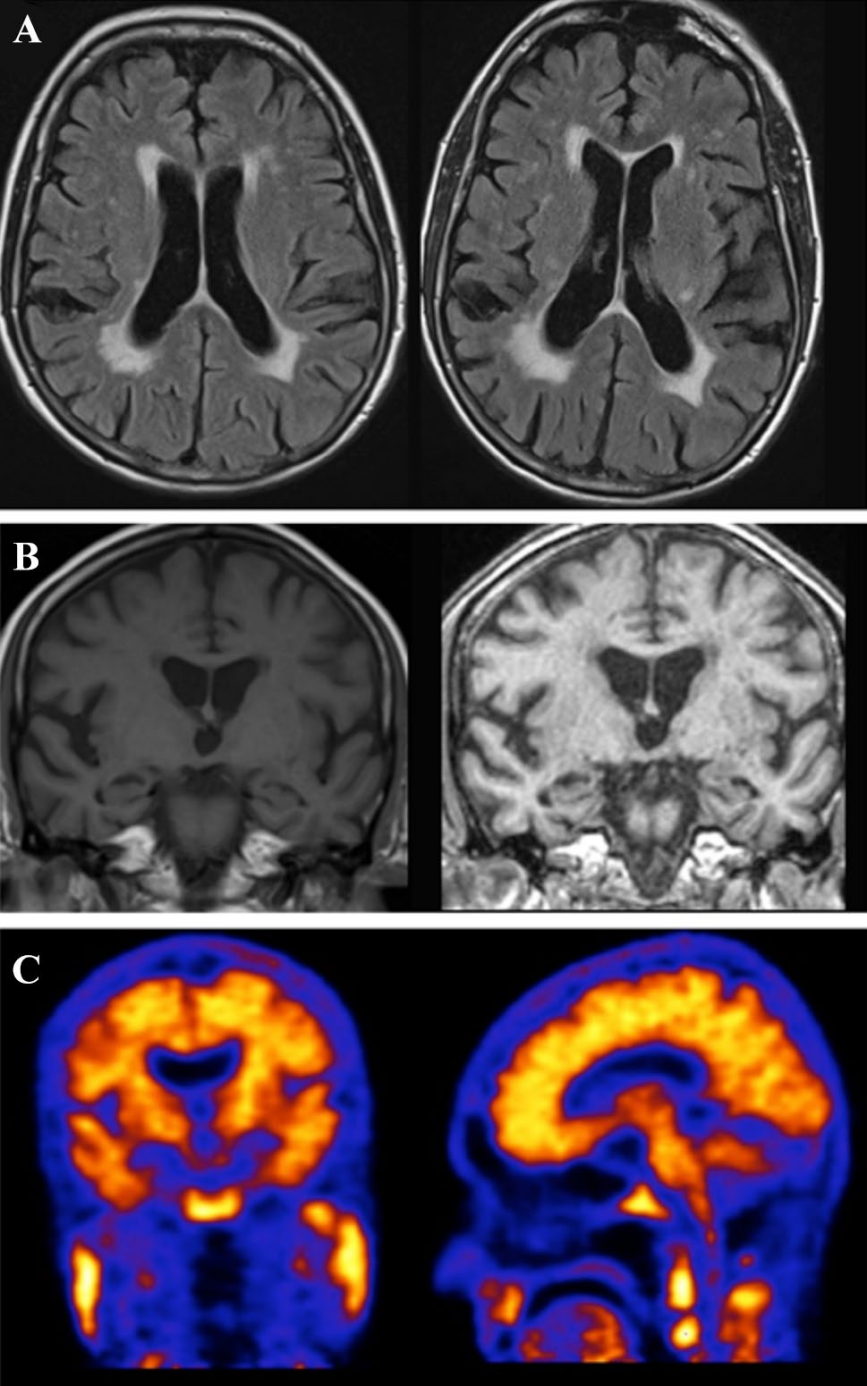
Case 3. Axial FLAIR images through the brain (**a**) demonstrating white matter changes over a 3-year interval. Coronal T1- and T2-weighted images through the brain (**b**) showing progressive atrophy of the brain over the 3-year interval. The most recent scan (right) shows bilateral medial temporal lobe atrophy, more so on the right. Amyloid PET imaging (**c**) was positive with loss of grey–white differentiation consistent with widespread amyloid deposition

A woman was referred with a 2-year history of progressive amnesia and concentration problems that had begun in her late 60s. She had a history of relapsing–remitting MS, diagnosed in 2000. In 2008, she was diagnosed with polymyalgia rheumatica for which she had been given Prednisolone followed by steroid-sparing agents (Azathioprine and Mycophenolate Mofetil, then Methotrexate). ACE-III score was 87/100, and neuropsychological assessment demonstrated impairments in executive function, memory (particularly encoding of new information) and processing speed. MRI brain showed confluent white matter changes on MRI, but no significant change in T2 lesion load when compared to previous imaging (Fig. 3a). There was mild generalised cerebral volume loss, more prominent in both temporal lobes (Fig. 3b). API, carried out after MDT discussion, was positive (Fig. 3c) and a diagnosis of AD was made. Donepezil was commenced with some initial improvement in symptoms. However, in the longer term, she continued to deteriorate cognitively with functional consequences, although her MS remained stable.

## Discussion

Cognitive impairment in MS may be related to mood, fatigue and sleep disturbance as well as strategic lesions [2, 7]. Using clinical assessment and structural imaging, it is often difficult to differentiate degeneration related to MS progression from coexisting Alzheimer’s, with the underlying diagnosis only becoming clear following post-mortem examination [18]. In such situations, API offers non-invasive detection of beta-amyloid plaques, enabling recognition of AD in the MS population and earlier introduction of appropriate management.

Here, three MS patients, all in their 70s, presenting with progressive cognitive impairment of unclear aetiology were described. Following MDT discussion, they underwent API because of a lack of diagnostic clarity from standard investigations. One patient had a negative amyloid PET scan, while two were diagnosed with AD based on positive scans. Both individuals diagnosed with AD were previously thought to have MS-related cognitive decline. API led to a change in diagnosis, as well as a change in management, including initiation of new medication and enrolment into clinical trials. Importantly, patients and their families could also be provided with information about the prognosis and appropriate support services available to them. This is in keeping with recent studies examining the wider utility of amyloid PET imaging [5, 13]. When used in individuals who meet appropriate criteria [8], API reduces the number of further investigations and significantly affects clinical management, in addition to increasing diagnostic certainty.

In MS, which is associated with cortical atrophy [17], examination of structural imaging for typical AD atrophy patterns is challenging as the combination of MS- and AD-related cortical atrophy leads to a less well-defined pattern. This is illustrated by Cases 1 and 3, where both patients had a degree of asymmetrical hippocampal atrophy. This is a relatively typical finding in Alzheimer's disease and strongly associated with a positive amyloid PET [1], but regional hippocampal atrophy has also been found in patients with MS [16], with some reports also describing asymmetry [15]. Thus, API may provide key information regarding the underlying cause of cognitive decline. CSF examination for amyloid β1-42 and Tau levels is a possible alternative approach but is invasive with more potential for adverse effects. Moreover, establishing CSF cut-off points is difficult, as evidenced by the wide variation in normal ranges used in different centres. Furthermore, there is evidence that atypical AD syndromes may have less clear-cut CSF profiles than are normally observed in typical AD [12].

One potential issue when using API in MS is that the reduced tracer uptake that has been found in demyelination might result in a failure to detect clinically relevant amyloid plaque, generating false-negative results. A recent study found that cortical β-amyloid deposition measured with API was lower in ageing MS patients than the controls matched for age, sex and APOE ε4 status [20]. While the authors concluded that MS could be protective of beta‐amyloid pathology, this finding may also reflect a loss of tracer binding due to demyelination, resulting in a falsely low estimation of cortical amyloid accumulation. Importantly, the research cohort imaged in this study mainly comprised cognitively unimpaired MS patients, in contrast to the case series described here, all of whom had dementia (and in whom ApoE status was not tested).

Another potential concern is that the proportion of cognitively normal individuals with clinically silent amyloid PET increases with age, and that any amyloid deposition may not be responsible for cognitive symptoms. However, these patients’ subsequent clinical course was in keeping with the diagnoses made with API.

Furthermore, although AD pathology seems to develop in MS in a similar incidence to that observed in normal ageing [6], it may be that individuals with MS are more vulnerable to the effects of amyloid because of their pre-existing cortical atrophy, leading to a shorter asymptomatic phase of AD. Studies such as that by Zeydan and colleagues which use modalities that are currently available in the research setting, such Tau PET imaging, may shed further light on this. However, given the possible confounds associated with tracer binding, neuropathological studies are more likely to be definitive.

In clinical practice, implementation of API in appropriate MS patients is recommended through a multidisciplinary approach according to appropriate use criteria in order to aid diagnosis and management of patients with cognitive decline. As described here, it may provide diagnostic clarity and assist with therapeutic decisions, while reducing the overall burden of investigations.
